# Bioelectromagnetism in Human Brain Research: New Applications, New Questions

**DOI:** 10.1177/10738584211054742

**Published:** 2021-12-07

**Authors:** Joachim Gross, Markus Junghöfer, Carsten Wolters

**Affiliations:** 1 Institute for Biomagnetism and Biosignalanalysis, University of Münster, Münster, Germany

**Keywords:** Bioelectromagnetism, MEG, EEG, TMS, TES, neurostimulation, electrophysiology, Helmholtz reciprocity, magnetoencephalography, electroencephalography

## Abstract

Bioelectromagnetism has contributed some of the most commonly used techniques to human neuroscience such as magnetoencephalography (MEG), electroencephalography (EEG), transcranial magnetic stimulation (TMS), and transcranial electric stimulation (TES). The considerable differences in their technical design and practical use give rise to the impression that these are quite different techniques altogether. Here, we review, discuss and illustrate the fundamental principle of Helmholtz reciprocity that provides a common ground for all four techniques. We show that, more than 150 years after its discovery by Helmholtz in 1853, reciprocity is important to appreciate the strengths and limitations of these four classical tools in neuroscience. We build this case by explaining the concept of Helmholtz reciprocity, presenting a methodological account of this principle for all four methods and, finally, by illustrating its application in practical clinical studies.

## Introduction

To study the human brain, scientists often rely on non-invasive methods from bioelectromagnetism such as magnetoencephalography (MEG) and electroencephalography (EEG) as well as transcranial brain stimulation techniques. MEG is closely related to EEG and both afford noninvasive, multichannel measurements of neuronal activity at high temporal resolution in the order of milliseconds. They are therefore ideally suited to study brain dynamics. Excellent reviews provide comprehensive introductions to and overview of MEG and EEG and their applications (e.g., Baillet 2017; Biasiucci and others 2019; Brette and Destexhe 2012; Gross 2019; Hari and Puce 2017). Similarly, guidelines for the analysis of MEG and EEG signals and the report of such analysis results have been published (Gross and others 2013; Hari and others 2018; Keil and others 2014; Pernet and others 2018) and will not be covered here. Instead, we want to focus on a topic that has received much less attention so far—namely the theoretical and practical relationship between MEG/EEG and neurostimulation by means of transcranial electric (TES) or magnetic (TMS) stimulation.

Theoretically, both sets of techniques are fundamentally linked by the Helmholtz reciprocity theorem that we will describe in more detail below (Fernández-Corazza and others 2020; Helmholtz 1853; Nolte 2003; Rush and Driscoll 1969; Vallaghé and others 2009; Wagner and others 2016b; Wolters and others 2004). Through consideration of this theorem, we will see that MEG/EEG on one hand and TMS/TES on the other hand are different sides of the same coin as they are governed by the same physical principles. Practically, as tools in the hand of a clinical or cognitive neuroscientist, this close relationship becomes clear through their complementary roles: MEG/EEG allows the recording of neural activity whereas TMS/TES allows the manipulation of neural activity. In their respective roles, all these techniques are ultimately based on the same physical laws—the Maxwell equations.

In the following sections we will lay out the close relationship between MEG/EEG and TMS/TES from theoretical principles to their practical implications. We will start with a short introduction to these methods, then describe the importance of their close relationship for forward and inverse modeling (see Box 1 for a definition of these terms) and finally discuss practical examples for their synergistic use in basic cognitive and clinical applications.

**Box 1**

**Helmholtz Reciprocity**

**Figure 1.**
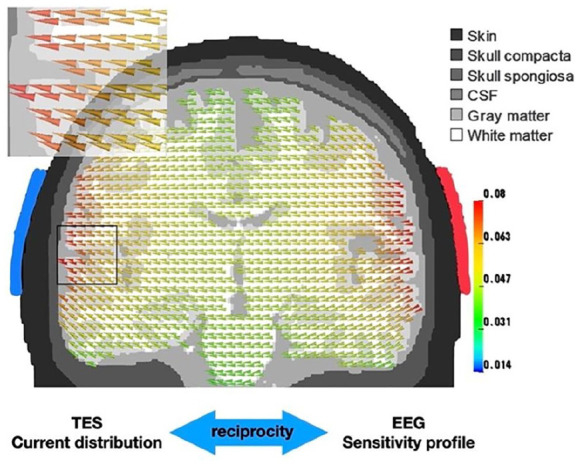
Illustration of the Helmholtz reciprocity theorem. The same vector field represents, on one hand, the current distribution induced by two transcranial electrical stimulation (TES) electrodes (red patch: anode, blue patch: cathode) and on the other hand, the sensitivity profile of these two electrodes to current sources at any given location.

At the heart of the intimate relationship between MEG/EEG and TMS/TES lies the reciprocity theorem that dates back to Helmholtz (Helmholtz 1853). While it is based on fundamental laws of physics and has significant consequences for the practical use of these techniques, the underlying idea is relatively straightforward. Here, we introduce this theorem in the context of TES and EEG. Consider a simple TES scenario where currents are injected and discharged via two electrode patches over right (anodal red) and left (cathodal blue) temporal brain areas. Figure 1 shows the calculated distribution of current intensity and current orientation in the brain, computed in a three-compartment (skin, skull, brain) head model. Importantly, the same distribution has a second equally valid interpretation as it represents the sensitivity profile (called leadfield) of an EEG recording with this particular electrode configuration (just two electrodes). More precisely, the vector in a certain voxel (described by position, orientation, and magnitude/length) illustrates which potential difference between the two electrodes would be measured if an assumed neuronal source with a unit strength of 1 was oriented in the direction of the cone. For a current of any orientation the projection onto this leadfield vector can be computed to yield the measured potential. A current oriented perpendicular to the cone orientation would for instance not generate a measurable difference potential between the two electrodes (projection equals zero).

Thus, neural sources in subcortical regions (dark green) are more difficult to excite via transcranial direct current stimulation/transcranial alternating current stimulation (tDCS/tACS) than cortical structures which are located closer to the tDCS patches (red) and neural generators in deeper structures reciprocally evoke smaller potential differences between the electrodes. Although deeper structures are excitable by stronger currents only, this stimulation would in parallel excite superficial sources to a much stronger degree unless sophisticated interference techniques are used (Grossman and others 2017). Similarly, MEG/EEG are less sensitive to neural activity from subcortical brain areas (Piastra and others 2021) and the localization of these sources is less accurate compared with cortical sources. Another important reciprocity in particular concerns MEG and TMS: As sources which are predominantly oriented orthogonal to the scalp (i.e., radial sources) evoke almost no measurable magnetic fields outside the head, these sources can, reciprocally, not get excited or inhibited via magnetic stimulation.

The concept of Helmholtz reciprocity is related to the concepts of forward and inverse problems that are equally relevant for the remainder of this manuscript. The forward problem in the context of EEG/MEG refers to the problem of estimating the potentials and/or magnetic fields at EEG electrode and/or MEG sensor locations on or/and outside the head for a dipole at a specific location in the brain with a given orientation. The inverse problem refers to the problem of estimating the current source distribution in the brain that gives rise to a given potential or/and magnetic field pattern on or/and outside the head. It was already noted by Helmholtz that the solution to the EEG/MEG inverse problem is not unique—that is, an infinite number of current distributions can lead to the same potential or/and magnetic field pattern. Therefore, EEG/MEG source localisation methods employ additional constraints to yield a unique solution.

## MEG/EEG and TMS/TES

MEG/EEG sensors silently and non-invasively sample with millisecond temporal resolution the magnetic field or electric potential, respectively, that is caused by neuronal activity in the brain. The direct relationship between the recorded signals and the underlying neuronal currents is not affected by intermediate processes (such as neurovascular coupling) and thereby leads to an information-rich dynamic representation of large-scale neuronal activity. While MEG/EEG perform non-invasive recordings of signals generated by neuronal activity, the aim of TMS/TES is the controlled modulation of neuronal activity by means of injecting magnetic fields or electric currents into the brain (Antal and others 2017; Polanía and others 2018).

There are several advantages in combining MEG/EEG with neurostimulation techniques (Bergmann and others 2016; Polanía and others 2018). First, it allows researchers to assess and monitor the effect of neurostimulation on neural activity, by utilizing the full-brain coverage and high temporal and good spatial resolution that MEG/EEG offers. Second, the neurostimulation setup can be optimized for specific targets (Fernández-Corazza and others 2020) and modulate specific neural activity patterns (such as brain oscillations) to probe their (causal) relevance for cognitive processes (Herrmann and others 2013; Thut and others 2017). Third, neurostimulation has significant translational relevance, particularly for treating neurological and mental health disorders (Antal and others 2017; Giovanni and others 2017; Tremblay and others 2019). Here, MEG/EEG and neurostimulation can be combined to help optimize stimulation protocols, identify mechanisms of action, and to more robustly assess treatment effects.

MEG and EEG signals can even be simultaneously recorded during TMS/TES with the current exception of simultaneous TMS/MEG. While EEG recordings can momentarily be interrupted during phasic TMS, preventing EEG amplifier saturation (Thut and others 2017; Tremblay and others 2019), this option awaits the new generation of MEG sensors (see Box 2). Though not as strong as TMS, TES also generates quite strong artefacts during EEG and MEG recordings, but modern EEG amplifier and modern SQUID (superconducting quantum interference device) sensors can tolerate currents that are typically applied (<4 mA) (Herring and others 2019; Marshall and others 2016; Ruhnau and others 2016; Witkowski and others 2016). However, removing TES/TMS artefacts from the EEG and MEG signals is not trivial because the amplitude of the artefact is modulated by a number of rhythmic and non-rhythmic processes, such as heartbeat, respiration, head movement, and changes in electrode impedance (Neuling and others 2017; Noury and Siegel 2018).

**Box 2**

**Optically Pumped Magnetometers: New Magnetoencephalography (MEG) Technology**

**Figure 2.**
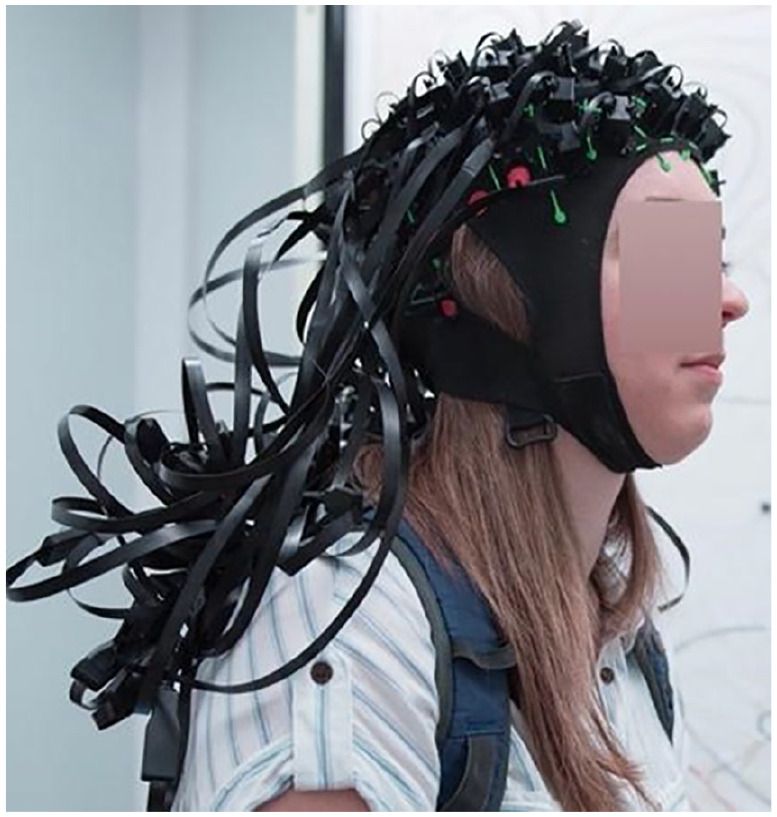
Example of a 50-channel optically pumped magnetometer (OPM) system (figure courtesy of Matt Brookes).

Optically pumped magnetometers (OPMs) have been developed recently and represent a promising alternative to traditional SQUID-based MEG systems (Labyt and others 2019). OPMs do not rely on superconductivity to operate and therefore do not require liquid helium. As a consequence, OPM-based MEG systems are easier and cheaper to maintain. A typical design uses a photodiode to measure the intensity of laser light after it has passed through a gas-filled glass cell (Boto and others 2018). Light transmission is sensitive to changes in the ambient magnetic field which can be detected by the photodiode. The sensitivity of OPMs has significantly increased in recent years and is now similar to the sensitivity of SQUID sensors. The size of OPM sensors could also be significantly reduced and therefore can now be integrated in mobile systems—similar to EEG (see Fig. 2). Importantly, OPMs benefit from the reduced distance between sensors and the brain leading to a comparable performance of current OPM systems (with less than 50 sensors) to SQUID systems with more than 100 sensors (Hill and others 2020; Iivanainen and others 2020). Despite their obvious advantages OPMs are limited by a relatively low signal bandwidth (about 150 Hz compared with several kHz for SQUIDs) and several technical challenges (such as crosstalk between neighboring sensors) which need to be addressed for high-density whole-scalp OPM systems.

**Figure 3.**
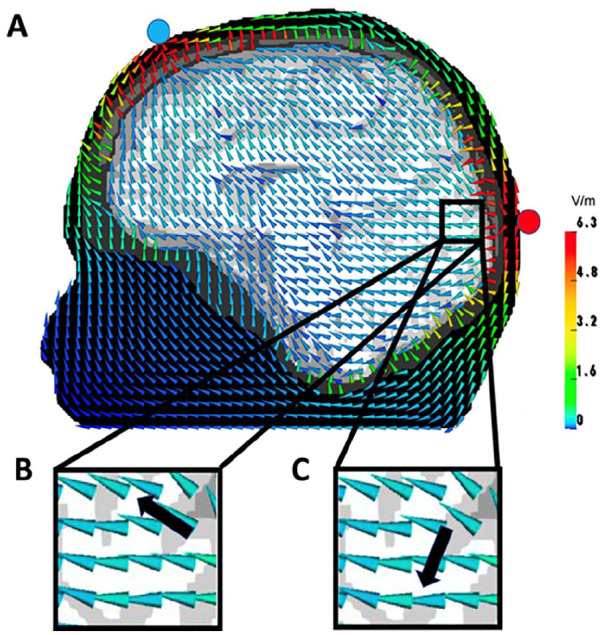
(A) Transcranial electrical stimulation (TES) electric field for an occipital anode (red circle) and a frontal cathode (blue circle) computed in a six-compartment (skin, skull, compacta, skull spongiosa, cerebrospinal fluid, gray matter, and anisotropic white matter) finite element model. Size-normalized cones are used to present vector orientation and they are color-coded to present vector field amplitudes. According to Helmholtz reciprocity, the electroencephalography (EEG) potential difference between the two electrodes of a dipole source (black arrow) can be determined by the dot product between the dipole moment and the electric field vector at the same position, so that scenario (B) would produce a maximal and (C) a zero potential difference.

Another important consideration for EEG/MEG-TES studies is the optimization of the stimulation parameters, including TES electrode location (Fernández-Corazza and others 2020; Opitz and others 2018; Wagner and others 2016a). Stimulation of a specific target area or a target network can be improved by the use of computational models that are based on realistic volume conductor models (Fernández-Corazza and others 2020; Huang and others 2017; Wagner and others 2014) ideally derived from individual anatomical magnetic resonance imaging (MRI), computed tomography (CT), and so on (Liu and others 2018) and/or individual functional measures based on EEG/MEG, functional MRI (fMRI), positron emission tomography (PET), near infrared spectroscopy (NIRS), and so on (see Fig. 3). Modern multichannel TES systems offer further degrees of freedom to control the path, focality and orientation of induced currents to optimally stimulate a target area (Baltus and others 2018). This is a promising and active research area driven by the exciting prospect of combining spatiotemporally detailed electrophysiological recordings with a versatile neurostimulation technique.

While simultaneous TMS-MEG is not currently possible, TMS has been used in conjunction with MEG by exploiting longer-lasting stimulation effects (i.e., after effects; e.g., Cao and others 2017). In these types of studies, TMS is used to stimulate the target area with an excitatory or inhibitory TMS protocol that is supposed to lead to sustained changes in neural activity that can persist for several hours. The behavioral and neural effects can, for instance, be assessed by comparing two MEG sessions before and after stimulation (Notzon and others 2018), an approach that can be used for any neurostimulation technique.

In the next section, we discuss the intricate relationship between MEG/EEG and TMS/TES from a methodological perspective.

## The Inverse Problem in EEG and MEG: Source Analysis

Over the past decades, EEG and MEG source analysis have become a prominent technique for reconstructing neuronal networks with highest temporal and appropriate spatial resolution (Baillet and others 2001; Grech and others 2008; Michel and others 2004). The so-called EEG and MEG inverse problem aims at reconstructing the sources (Murakami and Okada 2006)—predominantly in the brain gray matter—that are underlying measured potential and field distributions at the head surface. It has already been shown (Helmholtz 1853) that, without any additional prior on the sources, the solution to the inverse problem is not unique—that is, an infinite number of source distributions inside the volume conductor can lead to one and the same electric potential and/or magnetic field pattern outside the volume conductor. Therefore, EEG and MEG source localization methods employ additional constraints on the underlying source distribution to yield a unique inverse solution. A classification of these inversion methods could be made into focal current modeling, beamforming, and distributed current modeling. In focal current modeling, a small number of dipolar sources are fitted to the measured EEG and/or MEG data (Mosher and others 1992; Scherg and Von Cramon 1985; Wolters and others 1999). The first of the below-presented applications exemplifies such a focal current modeling result (the target black cone in Fig. 6) at the onset of averaged EEG and MEG spike activity of an epilepsy patient discussed in more detail below. When the number of sources is unknown or the current distribution might have a larger spatial extent, focal current models are not suitable. Spatial filtering or beamforming methods, as for instance used in the second example to estimate neural sources during the swallowing act (see Fig. 7), optimize the estimate at a single location or a small region while suppressing crosstalk from other areas (Gross and others 2001; Van Veen and others 1997). In distributed current models, the current is discretized by a large number of focal elementary sources having a fixed location and possibly also orientation. This approach, which has for instance been applied in the source reconstruction of emotional face processing in the following third example (see Fig. 8), is called current density reconstruction. Then, a priori information on the global properties of the solution is incorporated, for example, minimum norm estimation (MNE) (Hämäläinen and Ilmoniemi 1994). It should be mentioned that hierarchical Bayesian modeling forms a superclass of several inversion methods (Rezaei and others 2020). Importantly, all inverse methods can be used to identify the location and orientation of activated neuronal populations that can be defined as targets for neurostimulation.

## The Inverse Problem in Targeted Multichannel Brain Stimulation

Brain stimulation techniques such as TES allow modulation, that is, activation or inhibition, of neural activity and functional connectivity within brain networks. TES, which subsumes transcranial direct (tDCS) or alternating current stimulation (tACS), is a noninvasive method to manipulate brain excitability via changes in membrane polarization and to induce long-lasting (minutes or even hours) changes in the brain, depending on polarity, duration, and intensity of the stimulation (Antal and others 2017; Herrmann and others 2013). In classical tDCS, a small current (e.g., 0.5-4 mA) is applied to the human head by at least one electrode (anode) and removed at another electrode (cathode). Figure 3A shows a simulation of such a classical tDCS setup. This current can increase or decrease cortical excitability in the regions of interest, depending on its polarity (Antal and others 2017). Traditional bipolar tDCS setups assume that so-called “anodal stimulation” increases the excitability within the underlying cortical area (in Fig. 3A, the occipital area). However, recent studies demonstrated that the cortical current flow pattern in such setups is rather broad with maximal stimulation often in non-target brain regions and that not only target location is relevant for the optimal stimulation but also target orientation (Krieg and others 2013; Schmidt and others 2015; Wagner and others 2016a). Target orientation here refers to the dominant spatial orientation of neurons in the brain area targeted for neurostimulation. Injecting currents with tDCS along this dominant direction is important for maximal stimulation effect. For example, in cortical areas pyramidal neurons are often oriented perpendicular to the cortical surface. In subcortical brain structures such as hippocampus and amygdala, a preferred orientation of neurons is often absent rendering the concept of target orientation meaningless. For example, Mills and colleagues showed the sensitivity to both target location and orientation using different TMS coil orientations over the hand representation in primary motor cortex, while recording motor-evoked potentials (MEPs) at the contralateral hand muscle (Mills and others 1992). Their results indicate that the direction of current flow is critical, that is, not only is the position of the important but also its orientation relative to the brain and the direction of current flow resulting from it, with the largest MEP responses for a coil at about 50° to the parasagittal plane, producing a maximal induced current flowing forward approximately at right angles to the central sulcus. In a similar experiment using TMS and MEP coupled with tDCS, Rawji and others (2018) showed that the direction of current flow is important for tDCS after effects as well. Therefore, a tDCS stimulation setup as illustrated in Figure 3A would for example generate an electric field inside the brain that is predominantly oriented parallel to a target (black arrow) with location and orientation as displayed in scenario (B). Thus, this specific electrode setting would have a strong excitatory effect on this specific parallel oriented target. However, for a target with almost identical location but now orthogonal orientation to the electric field as displayed in Figure 3C, stimulation with this electrode configuration would have almost no effect (i.e., the dot product between the electric field vector and the target moment would be almost zero). An appropriate targeting thus means that (1) the injected current should not only be maximal at the area of interest (intensity) and (2) minimal at other areas (focality) but also (3) predominantly oriented parallel (excitation) or anti-parallel (inhibition) to the target orientation (directionality). Because of the complexity of such targeting and the up-coming multichannel tDCS (mc-tDCS) hardware, computer optimization approaches, that is, novel methods for the solution of the mc-tDCS inverse problem have become important tools for targeted brain stimulation (Dmochowski and others 2011; Wagner et al., 2016a). Recently, a unification of such optimization approaches has been derived and a focality-intensity trade-off has been shown (Dmochowski and others 2011; Fernández-Corazza and others 2020; Khan and others 2021), that is, optimization methods can be sorted on a focality-intensity scale with focal approaches on one side of the scale and intensity-based optimization methods on the other side, illustrating that focality and maximum stimulation intensity at the target cannot be achieved with one and the same method. Finally, with regard to stimulation intensity, Agboada and others (2019) showed that it is nonlinearly related to stimulation outcome, which might be associated with intracellular calcium increases: Larger stimulation intensity increases calcium levels to induce long-term potentiation (LTP)-like plasticity, while lower stimulation intensity resulted in long-term depression (LTD)-like plasticity.

Please note that the simple scenario where injecting currents parallel to target orientation leads to excitation and anti-parallel leads to inhibition holds for tDCS only. Other stimulation methods and protocols will engage other mechanisms of action. For example, tACS employs electric stimulation with rapidly alternating current direction. rTMS applies single pulses in rapid succession. Both methods are thought to lead to frequency-specific resonance effects that may be used to change the amplitude of neuronal rhythms (Thut and others 2017).

## Helmholtz Reciprocity, the Bioelectromagnetic Forward Problem, and Head Modeling

**Figure 4.**
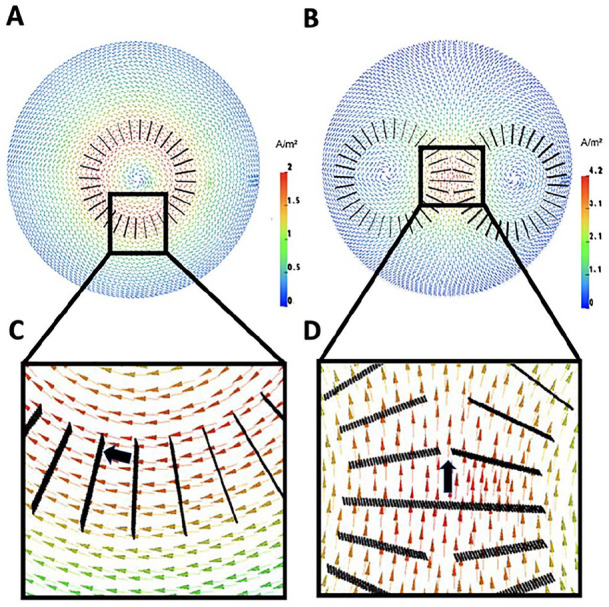
Transcranial magnetic stimulation (TMS)-induced current density vector field for a circular coil (A) and a figure-of-eight coil (B) computed in a tetrahedral multicompartment sphere finite element model, visualized on a cut-plane through the model. According to Helmholtz reciprocity, the magnetoencephalography (MEG) magnetic flux for a dipole source (black arrow) at a (circular) magnetometer coil (C) and the (figure-of-eight) tangential gradiometer (D) follows directly from the dot product between the dipole moment and the field vector at the same position in the volume conductor.

**Figure 5.**
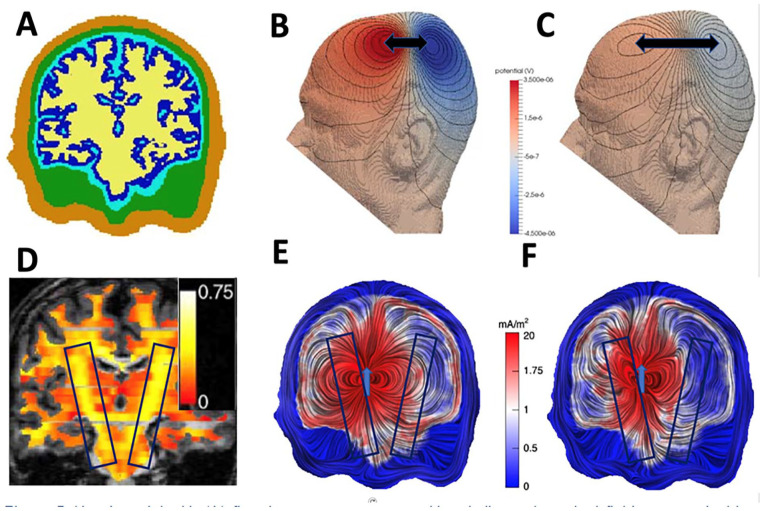
Head model with (A) five tissue compartments skin, skull, cerebrospinal fluid, gray matter, and white matter. (B, C) Isopotential lines for a mainly tangentially oriented source in somatosensory cortex where the underlying model in (B) has 10 times higher skull conductivity than in (C). The resulting smaller distance between potential peak and trough in (B) as compared with (C) are indicated by black arrows. (D) fractional anisotropy based on diffusion tensor magnetic resonance imaging (MRI) for the white matter compartment. Volume currents for a thalamic dipole source in the head model with (E) isotropic and (F) 1:10 anisotropic white matter conductivity anisotropy (reprinted from Wolters and others 2006) Copyright, with permission of Elsevier).

To improve performance, it can be vital to couple the aforementioned inverse and optimization approaches with modern forward modeling methods. The EEG and TES as well as MEG and TMS forward problems are closely related through Helmholtz’s reciprocity principle (Fernández-Corazza and others 2020; Helmholtz 1853; Nolte 2003; Rush and Driscoll 1969; Vallaghé and others 2009; Wagner and others 2016b; Wolters and others 2004), which means that any accuracy improvement of one of these will reciprocally lead to an improvement of the other one, too. For the reciprocity of TES and EEG, this is visualized in Figure 3 and for the reciprocity of TMS and MEG in Figure 4. More explicitly Wagner and others (2016b) were able to show that the Helmholtz reciprocity relation offers a direct link between the TES and EEG forward problems by analyzing numerical results, comparing to analytically derived forward potentials in simplified volume conductors, estimating computational complexity and even deriving an algebraic proof valid even for realistic head volume conductor models. For the magnetic forward problem, such a reciprocity-based relationship has been worked out by Nolte (2003) and Vallaghe and others (2009). Therefore, in the following, we will merge the bioelectromagnetic forward problems of source analysis on one hand and the simulation of non-invasive brain stimulation on the other hand in our presentation. In contrast to the EEG inverse problem, existence and uniqueness of the solution to the EEG forward problem have been proven (Wolters and others 2007). Depending on the available input data, different forward modeling approaches have been proposed, from quasi-analytical solutions for simplified multilayer sphere head models for EEG (de Munck and Peters 1993) and overlapping spheres for MEG (Huang and others 1999) to realistically shaped head models with one compartment for MEG (Nolte 2003) or three isotropic compartments (3CI: skin, skull, brain), for EEG in combination with the boundary element method (BEM) (Kybic and others 2005) or the finite element method (FEM) (Piastra and others 2021). Comparisons between different forward modeling approaches allow not only validation of one method against the other, but especially also to determine model error and numerical error as well as computational performance (Htet and others 2019; Medani and others 2021; Piastra and others 2021; Vorwerk and others 2012). Although 3CI head models geometrically represent the skull and skin surfaces individually and accurately and thus already reduce model errors in comparison with simpler multilayer sphere models, they are still based on a quite rough homogenization and approximation of head volume conduction (i.e., electrical conductivities within the different tissues). First of all, in standard 3CI modeling, most often standard literature values are used for the electrical conductivity parameters. However, the specific importance of correct skull conductivity modeling for the bioelectric forward problems has been shown for EEG (Akalin Acar and others 2016) and TES (Antonakakis and others 2020; Saturnino and others 2019; Schmidt and others 2015; Vorwerk and others 2019), and it is known that skull conductivity varies significantly across individuals and can be estimated from non-invasive neurophysiological data (Antonakakis and others 2020; Saturnino and others 2019; Vorwerk and others 2019). Figure 5 exemplifies the relevance of accurate skull conductivity modeling.

A tangentially oriented source located in the somatosensory cortex of a 5-compartment head model (A) built of skin, skull, cerebrospinal fluid (CSF), gray matter, and anisotropic white matter (D) would generate EEG isopotential lines on a model surface as shown in (B) and (C). The only difference between B and C is that skull conductivity was modelled ten times higher in (B) compared to (C) leading to much higher surface potentials and a smaller distance between the positive potential peak and the negative potential trough in (B) compared to (C), as indicated by the black arrows. In the context of the EEG inverse problem, ignoring the variance of individual skull conductivities would thus predominantly lead to depth localization errors (as the distance between peak and trough increases with source depth). Apart from skull conductivity modeling, the importance of accurate CSF conductivity modeling has been shown not only in simulations (Piastra and others 2021; Wagner and others 2014) but also experimentally (Rice and others 2013). Figure 5E and F, for instance, illustrates the impact of the high CSF conductivity on volume currents as generated by a thalamic source leading to higher amplitude volume currents and current channeling in the CSF compartment and therefore to reduced EEG surface potential magnitudes (Piastra and others 2021; Rice and others 2013; Wagner and others 2014). Finally, distinguishing between gray and white matter conductivity and modeling white matter conductivity anisotropy can further increase the accuracy in bioelectromagnetic forward modeling (Haueisen and others 2002; Vorwerk and others 2014). Here, and also as in Figure 3, the term *anisotropy* refers to the property of a material (here white matter) to allow changes in different directions in contrast to isotropy, that is, the conductivity parallel to the white matter tracts is higher than in the two perpendicular directions. To illustrate white matter conductivity anisotropy volume conduction effects, the lower row of Figure 5 shows volume currents for a thalamic dipole source with an isotropic (E) versus a 1:10 anisotropic (perpendicular:parallel to the white matter tracts) white matter compartment (F), visualized on a coronal cut through the models. Anisotropic white matter conductivity thus causes return currents to flow parallel to the white matter fiber tracts, which can be specifically observed by the larger influence of the highly anisotropic pyramidal tracts on the volume currents as indicated by black boxes in Figure 5D-F. Note also the corresponding high fractional anisotropy of the pyramidal tracts from diffusion tensor MRI in (D). It can be also concluded that the deeper the source, the more it is surrounded by anisotropic white matter tissue and the larger is the influence of anisotropy on the resulting fields.

Figure 3 shows a simulated TES electric field (EF) for an anode (occipital electrode) and a cathode (frontal electrode) in a six-compartment anisotropic (6CA: skin, skull compacta, skull spongiosa, CSF, gray matter and anisotropic white matter) FEM head model. The figure demonstrates important TES effects: First, mainly tangential EF orientations are found in the skin compartment. Second, EF orientation is mainly radial in the low conducting skull compartment. Third, the high CSF conductivity and the anisotropic white matter conductivity influence EF orientations in the compartments inside the inner skull surface considerably. Finally, EF amplitudes decrease mainly, but not only, with increasing distance to the stimulation electrodes.

Realistic individualized head models can be constructed using (semi-)automatic processing pipelines based on T1-weighted- (T1w-), T2w-MRI and diffusion-weighted-MRI (Antonakakis and others 2020; Huang and others 2019; Medani and others 2021; Nielsen and others 2018). This allows the use of realistic individualized head models for sample sizes typically used in cognitive and clinical neuroscience studies (e.g., Radecke and others 2020) with reasonable time investment and computing resources. Construction of realistic individualized head models and simulation of TMS/TES stimulation is available in open source toolboxes such as SimNIBS (Wolters and others 2006) and ROAST (Huang and others 2019). More specific open source toolboxes are on the topics of the bioelectromagnetic forward problems such as DUNEuro (Schrader and others 2021), OpenMEEG (Gramfort and others 2010), and BEM-FMM (Makarov and others 2021).

In the next section, we present four studies that combine MEG/EEG and TMS/TES and sample the wide spectrum of their synergistic use.

## Applications of Combining Neurostimulation with MEG/EEG

The number of studies combining high-density EEG/MEG neuroimaging with targeted TMS/TES brain stimulations increased exponentially in the past decade. Accordingly, the spectrum of applications in cognitive and clinical neuroscience has expanded considerably. The following four examples were picked to provide a glimpse on the range of methods, method combinations, and applications. This specific choice in part reflects the clinical orientation of the author’s research interests and is in no way intended to represent any kind of superiority over the multitude of excellent studies in the field.

Advances in functional neuroimaging continuously increase our understanding of pathological alterations in neural circuits that underlie brain disorders. This advanced knowledge leads to an increased interest in brain stimulation methods to modulate the identified aberrant neuronal activity patterns. Reciprocally, clinical treatment effects of brain stimulation can also inform us about underlying disease mechanisms and allow us to evaluate causal relations of stimulated brain areas in distributed networks going beyond purely correlational associations.

### Application of Combined tDCS/MEG/EEG for Testing Potential Novel Treatment Strategies for Pharmacoresistant Focal Epilepsy

EEG and MEG source analysis and connectivity investigations can, for instance, fundamentally contribute to the understanding of pathomechanisms underlying epileptogenesis, seizure generation, and seizure propagation (Adebimpe and others 2016; Aydin and others 2017; Rampp and others 2019). It may thus pave the way to new treatment options, including non-invasive (e.g., TES) (Kaufmann and others 2021; Yang and others 2020) and invasive forms like epilepsy surgery. While for some patients non-localized pathomechanisms must be assumed, others have a rather localized epileptogenic network, with a reasonable chance to become seizure-free after circumscribed cortical resections (Wellmer and others 2016).

**Figure 6.**
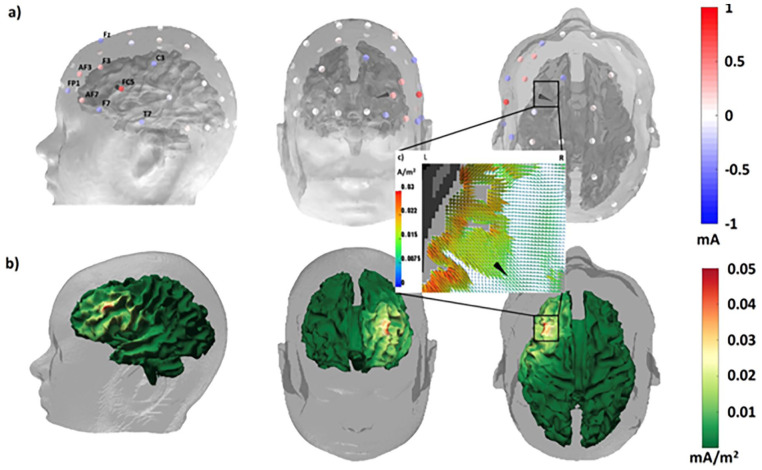
Individually targeted multichannel transcranial direct current stimulation (mc-tDCS) for inhibitory stimulation of a patient with pharmacoresistant epilepsy due to a focal cortical dysplasia located close to the Broca area. (a) Alternating direction method of multipliers (ADMM)-optimized mc-tDCS montage for stimulation of the epileptogenic zone as reconstructed by focal source modeling from electroencephalography (EEG) and magnetoencephalography (MEG) spike activity (black cone). The electrodes are colored by the optimized currents ranging from −1 up to +1 mA. The total sum of the injected currents is equal to 2 mA fulfilling safety constraints. The optimized mc-tDCS current density distribution visualized (b) on the cortical surface and (c) on a zoomed axial magnetic resonance imaging (MRI) view in the target area. The colored brain surface of the patient represents the distribution of the current density measured in mA/m^2^.

Individual targeting by optimized multichannel TES can improve therapeutic effects and decrease negative side effects. Figure 6 exemplifies an individualized therapeutic procedure for focal epilepsy: Inverse (focal current, see above) source modeling of the onset of averaged EEG/MEG spike activity was used to localize the epileptogenic zone (black cone in top row) of a pharmacoresistant focal epilepsy patient (Antonakakis and others 2019). A tDCS optimization with regard to both target location and orientation on the more focal side of the focality-intensity continuum (Dmochowski and others 2011; Fernández-Corazza and others 2020) resulted in a topography of injected and discharged currents that is visualized in the top row and evokes a focal, but low-intensity, current density distribution in the brain as shown in the bottom row. Because such focal stimulation reduces side effects, it could be applied more frequently and over a longer time period. A tDCS optimization aiming at maximum intensity in the target region would increase effect size but would also lead to stronger co-activation of surrounding regions potentially generating more side effects (Antonakakis and others 2019; Dmochowski and others 2011; Fernández-Corazza and others 2020; Radecke and others 2020). In this example, the combination of high-density EEG and MEG provided excellent target information serving as a prerequisite for highly focal multichannel stimulation targeting (see also Fig. 10) and thus illustrates how individualized tDCS brain stimulation may improve therapeutic outcome.

### Application of Combined tDCS/MEG for Testing Potential New Treatment Strategies for Dysphagia Resulting from Cerebral Stroke

Swallowing relies on highly complex sensorimotor functions requiring widely distributed neural network activities (Furlong and others 2004). It is thus not surprising that disordered swallowing (oropharyngeal dysphagia, OD) is a frequent complaint poststroke. Spontaneous recovery of OD has been related to compensatory changes in swallow-relevant areas of the unaffected hemisphere with enhanced cortical excitability and enlarged motor representation as surrogate of cortical plasticity.

**Figure 7.**
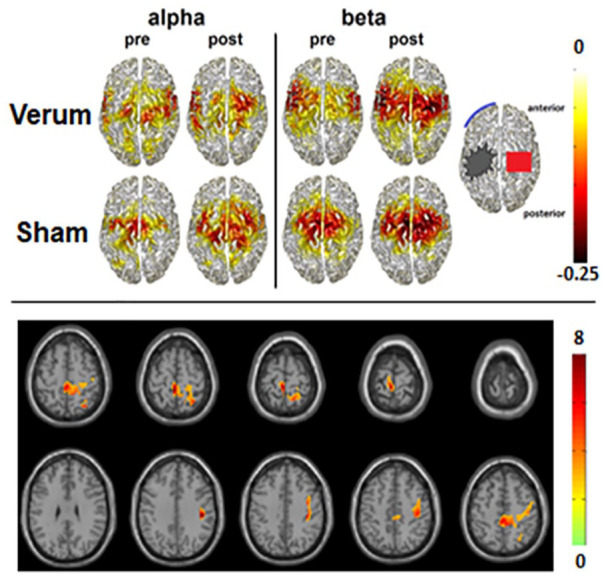
Source distribution of group mean swallowing-associated activation in the alpha and beta frequency range pre- and postintervention in the transcranial direct current stimulation (tDCS) verum and sham groups. The color bar indicates power changes relative to the resting stage. Negative values denote event-related desynchronization of oscillatory activity. Bottom: Areas with significant increase of swallow-related event-related desynchronization in the beta frequency range after real transcranial direct current stimulation (p < 0.05). Based on Suntrup-Krueger and others (2018).

In a randomized control trial, Suntrup-Krueger and colleagues revealed that excitatory tDCS stimulation of the unaffected motor cortical swallowing network resulted in greater improvement of swallowing functions compared with a sham stimulation (Suntrup-Krueger and others 2018). In fact, verum tDCS induced significantly greater improvement in the primary and secondary clinical outcomes than sham stimulation after 4 days of intervention. MEG measures before and after treatment performed in a subgroup of patients revealed that both groups showed an increase of event-related desynchronization (ERD) in the alpha and beta bands due to standard therapy (Fig. 7, top) but the verum group only revealed add-on effects in the beta band (Fig. 7, bottom). Thus, facilitated reorganization of swallowing network activity via excitatory tDCS accelerates rehabilitation of acute poststroke dysphagia.

This example illustrates that even non-individualized brain stimulation can modulate highly automatized and complex brain functions such as swallowing, holding out the prospect of standardized use in clinical practice.

### Application of Combined tDCS/MEG for Testing Potential Novel Brain Stimulation Targets for the Treatment of Mood and Anxiety Disorders

**Figure 8.**
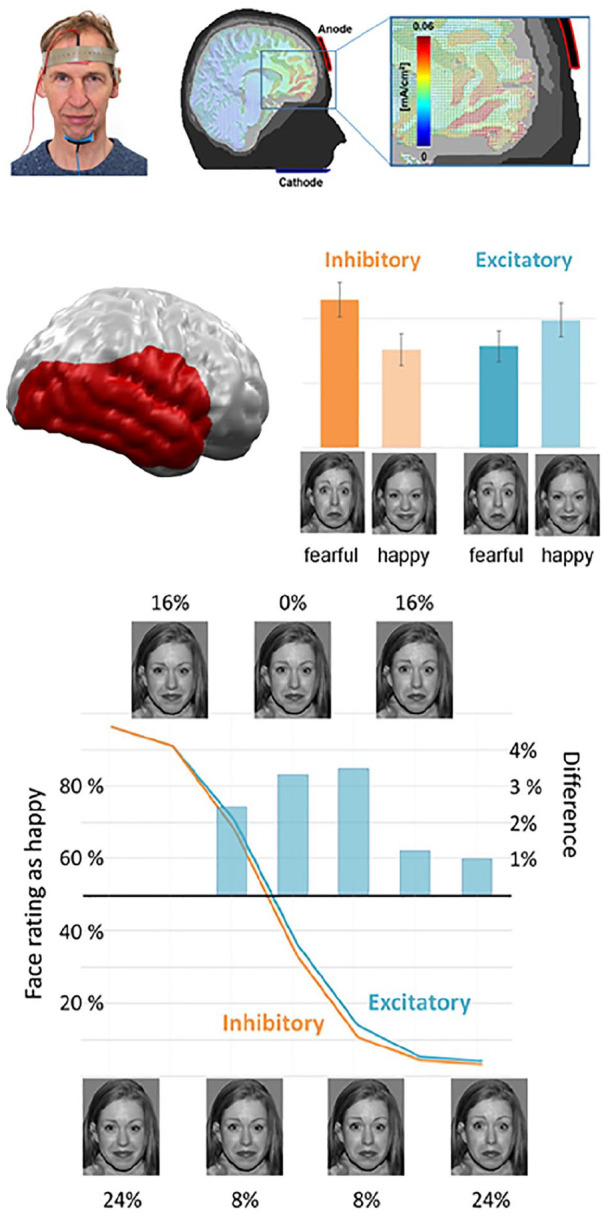
Excitatory (anodal) repeated transcranial direct current stimulation (tDCS) of the ventromedial prefrontal cortex (vmPFC) using an extracephalic reference led to a relatively enhanced processing of happy compared with fearful faces in the visual stream of the right hemisphere while inhibitory stimulation resulted in the reverse pattern. Accordingly, excitatory vmPFC stimulation enhanced the assessment of emotional ambiguous (morphed) facial expressions as happy. Based on Winker and others (2018).

The so-called ventromedial prefrontal cortex (vmPFC) is one of the most widely reported structures identified as dysfunctional in mood and anxiety disorders (Myers-Schulz and Koenigs 2012). In two independent fMRI and MEG studies, Junghofer and coworkers tested effects of a novel tDCS montage with an extracephalic reference for optimized vmPFC stimulation (see top of Fig. 8) and showed that excitatory relative to inhibitory stimulation amplified processing of pleasant compared with unpleasant emotional scenes in healthy participants (Junghofer and others 2017). In a follow-up study Winker et al. could replicate and generalize these findings to emotional face processing, as excitatory versus inhibitory vmPFC-tDCS led to an enhanced processing of happy compared with fearful faces consistent with an enhanced rating of happiness in ambiguous facial expressions (see bottom of Fig. 8) (Winker and others 2018). As excitation of the vmPFC seems to enhance appetitive relative to aversive stimulus processing, excitatory vmPFC-tDCS might ameliorate biases away from pleasant and in favor of unpleasant information as typically reported in patients suffering from mood disorders. The combination of tDCS and MEG could recently also reveal some novel insights into the potential causal role of the vmPFC in anxiety as inhibitory vmPFC stimulation induced “anxiety-like” perceptual and neural patterns of fear generalization (Roesmann and others, 2021). This example shows how neurostimulation in combination with MEG allows to test a presumed causal role of defined target regions for cognitive and affective processes potentially disturbed in neurological or psychiatric disorders. Regarding a further aspect of Helmholtz reciprocity, this example also illustrates how, for special targets, an extracephalic tDCS reference can circumvent the so-called “electric reference problem,” which is again mutually intrinsic for both, electric brain stimulation (tDCS) and electric recordings of brain signals (EEG).

### Application of Simultaneous TMS/EEG to Prove an Intrinsic Dysfunction in Thalamocortical Circuits in Schizophrenia

**Figure 9.**
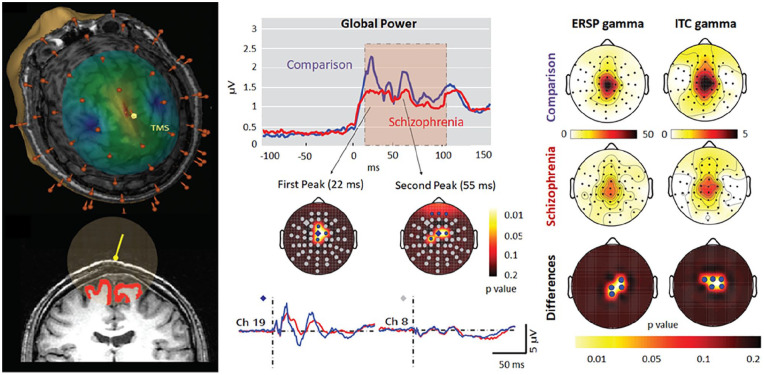
Left: Estimation of electric field intensity generated by transcranial magnetic stimulation (TMS) on the cortical surface and an estimate of the gray matter volume affected by TMS. Center: Timing and location of the reduced global field power of TMS evoked oscillatory activity in patients. Right: Topographies of reduced event-related spectral perturbation (ERSP) and intertrial coherence values in schizophrenia patients. Based on Ferrarelli and others (2008).

Schizophrenia patients typically show deficits in evoked gamma band activity and gamma synchrony at rest and during various cognitive tasks (e.g., Senkowski and Gallinat 2015). By taking advantage of a combined TMS/high-density EEG protocol, Ferrarelli and coworkers aimed at excluding a potential covarying impact of impaired motivation, attention, or cognitive capacity (Ferrarelli and others 2008). In fact, schizophrenia patients revealed aberrant gamma oscillations within the first 100 msec after TMS at a frontocentral region of stimulation (see Fig. 9, left column), which were significantly reduced in amplitude and synchronization (central column). Moreover, inverse EEG source modeling revealed that patients’ TMS evoked brain activation did not propagate away from the stimulated brain region. Since event-related EEG responses to direct cortical TMS are not affected by motivation, attention, or cognitive capacity and are not relayed through peripheral afferent pathways, these findings speak for an intrinsic dysfunction in thalamocortical circuits in schizophrenic patients. This example illustrates how a combination of neurostimulation and MEG/EEG allows a directed exclusion of potential covariates, which is in many cases much more difficult or even impossible using other, for instance correlational, methods.

## Discussion and Conclusion

The above examples gave an impression on how combinations of TMS/TES with MEG/EEG can produce synergistic effects. They also illustrated how different brain stimulation methods can be informed by MEG/EEG and then, for instance, be applied in the attempt to test potential novel treatment strategies or to uncover or prove pathological neural mechanisms. However, despite the increasingly successful use of targeted brain stimulation, knowledge about the mechanisms of action of the different brain stimulation methods, about their dependence on stimulation parameters such as duration, repetition, strength, frequency, and orientation, and about the modulation of stimulation effects by physiological and psychological states and characteristics is still rather scarce.

Here, we showed that due to Helmholtz’s reciprocity (Fernández-Corazza and others 2020; Helmholtz 1853; Nolte 2003; Rush and Driscoll 1969; Vallaghé and others 2009; Wagner and others 2016b; Wolters and others 2004), EEG and TES (Vallaghé and others 2009; Wagner and others 2016b) as well as MEG and TMS (Nolte 2003; Vallaghé and others 2009) are methodologically closely related to each other and the electric and magnetic modalities are complementary (Dassios and others 2007). For example, MEG is nearly blind to neural sources oriented toward the inner skull surface (radial), and these sources can also not be stimulated by TMS, while EEG is specifically sensitive to radial sources (Piastra and others 2021; Vorwerk and others 2014). While for radial targets standard 4 × 1 TES montages are focally stimulating and are thus on the focality side of the focality-intensity scale, the second standard of anode over the target and cathode far away maximizes target intensity and is thus on the intensity side of the scale (Dmochowski and others 2011; Fernández-Corazza and others 2020). MEG has higher sensitivity for tangential sources (Antonakakis and others 2020; Saturnino and others 2019; Vorwerk and others 2019), which can also be effectively stimulated with TMS (Krieg and others 2013; Schmidt and others 2015; Wagner and others 2016a). The complementary sensitivity profiles of MEG and EEG as well as tDCS and TMS thus motivate the combination of electric and magnetic modalities (Antonakakis and others 2019; Aydin and others 2017).

While we here focus mostly on the issue of reciprocity, it should be noted that long-lasting effects of brain stimulation that are exploited for therapeutic purposes, might not solely be related to direct modulation of targeted brain areas, but could also be caused by more general mechanisms, such as modulations of more distant areas and effects mediated by changes in neurotransmitters and gene expression (e.g., Chervyakov and others 2015; Diana and others 2017).

**Figure 10.**
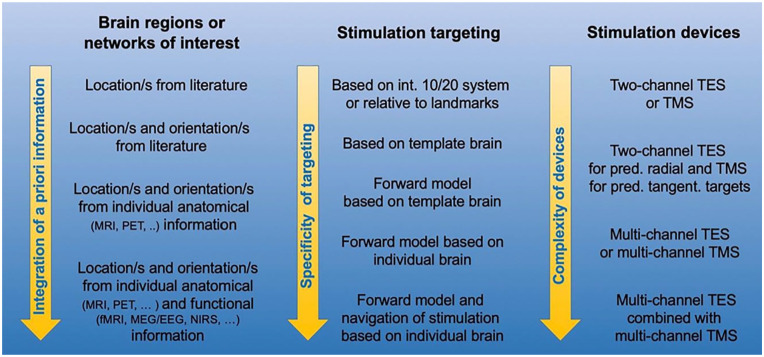
Illustration of a necessary mutual fit of a priori target information and complexity of stimulation targeting/devices. If target locations or target networks are just roughly known (e.g., ventral regions of the prefrontal cortex) standard targeting with basic TES/TMS devices is perfectly adequate while higher sophistication might even have detrimental effects. If individual target location and target orientations are very well defined (as in the above epilepsy example), best available targeting methods and stimulation devices should be applied.

Looking into the future, we expect that hardware developments and improved targeting procedures will increase the effectiveness of neurostimulation as scientists continue to strive to better control and understand the stimulation effects. It is now well established that modeling of stimulation effects (ideally with consideration of the individual anatomy) is important. However, stimulation effects depend on many factors that need to be considered together. Figure 10 illustrates this point by listing different levels of complexity or accuracy regarding the prior information about the target region of interest (left; e.g., inverse EEG/MEG modeling), the targeting procedure (center; e.g., forward modeling), and the stimulation devices (right). For example, multichannel TES optimization can improve the effectiveness of stimulation, but only if the individual target location and orientation is well known (e.g., see first above application example of focal epilepsy). Therefore, in the many situations where the stimulation target is more regional than focal or can only be roughly reconstructed, the standard two-patch TES procedure with anode over the target region and cathode far away (or even extracephalic) is reasonable, with high intensity if the target region has significant radial orientation components (e.g., see third application example of vmPFC stimulation). Therefore, the selection of stimulation devices and stimulation targeting procedures depend on the available prior information regarding the target. However, in all cases accurate report of stimulation parameters such as duration, intensity, coil/electrode location, and orientation is important to improve reproducibility and facilitate meta-analyses.

In summary, the complementary use of MEG/EEG and TMS/TES holds great potential not only for improving our understanding of cognitive processes in the brain but also for developing new therapeutic approaches for the treatment of neurological and psychiatric disorders.
